# GAP-43 closely interacts with BDNF in hippocampal neurons and is associated with Alzheimer's disease progression

**DOI:** 10.3389/fnmol.2023.1150399

**Published:** 2023-04-18

**Authors:** Ye Ji Lee, Ye Ji Jeong, Eun Ji Kang, Beom Seok Kang, Song Hee Lee, You Jin Kim, Seong Su Kang, Sang Won Suh, Eun Hee Ahn

**Affiliations:** ^1^Department of Physiology, College of Medicine, Hallym University, Chuncheon-si, Gangwon-Do, Republic of Korea; ^2^Department of Pathology and Laboratory Medicine, Emory University School of Medicine, Atlanta, GA, United States

**Keywords:** brain derived neurotrophic factor (BDNF), 7.8-DHF, growth-associated peptide 43 (GAP43), molecular interaction, hippocampal neuron, Alzheimer's disease

## Abstract

**Introduction:**

Growth-associated protein 43 (GAP-43) is known as a neuronal plasticity protein because it is widely expressed at high levels in neuronal growth cones during axonal regeneration. GAP-43 expressed in mature adult neurons is functionally important for the neuronal communication of synapses in learning and memory. Brain-derived neurotrophic factor (BDNF) is closely related to neurodegeneration and synaptic plasticity during the aging process. However, the molecular mechanisms regulating neurodegeneration and synaptic plasticity underlying the pathogenesis and progression of Alzheimer's disease (AD) still remain incompletely understood.

**Methods:**

Remarkably, the expressions of GAP-43 and BDNF perfectly match in various neurons in the Human Brain Atlas database. Moreover, GAP-43 and BDNF are highly expressed in a healthy adults' hippocampus brain region and are inversely correlated with the amyloid beta (Aβ), which is the pathological peptide of amyloid plaques found in the brains of patients with AD.

**Results:**

These data led us to investigate the impact of the direct molecular interaction between GAP-43 and BDNF in hippocampal neuron fate. In this study, we show that GAP-43 and BDNF are inversely associated with pathological molecules for AD (Tau and Aβ). In addition, we define the three-dimensional protein structure for GAP-43 and BDNF, including the predictive direct binding sites *via* analysis using ClusPro 2.0, and demonstrate that the deprivation of GAP-43 and BDNF triggers hippocampal neuronal death and memory dysfunction, employing the GAP-43 or BDNF knock-down cellular models and 5XFAD mice.

**Conclusion:**

These results show that GAP-43 and BDNF are direct binding partners in hippocampal neurons and that their molecular signaling might be potential therapeutic targets for AD.

## Introduction

Alzheimer's disease (AD) is the most common progressive neurodegenerative disease in the aging process. The major pathological hallmarks of AD are extracellular senile plaques, which are mainly composed of misfolded or aggregated polypeptide β-amyloid 42 (Aβ 42) and intraneuronal neurofibrillary tangles (NFTs), principally consisting of hyperphosphorylated Tau. These pathological changes in the development of AD occur decades before the onset of clinical symptoms. Synaptic dysfunction and plasticity loss are highly associated with the cognitive decline of AD (DeKosky and Scheff, 1990; Sze et al., 2000; Selkoe, 2002). Although huge efforts have been made in this research field, the key molecular mechanism dictating AD pathogenesis remains unclear. GAP-43 is a presynaptic secreted protein and is highly expressed during neuronal development and synaptogenesis thereafter in the hippocampus and the related cortices of the mature adult human brain (Neve et al., 1988; Suzanne et al., 1989). Moreover, GAP-43 is related to the regulation of neuron axonal outgrowth and synaptic plasticity in learning and memory (Aigner et al., 1995; Routtenberg et al., 2000; Young et al., 2000). Recent research reported that GAP-43 could be an early diagnostic marker for AD progression (Sandelius et al., 2019). BDNF, the neurotrophin ligand for TrkB receptors, is distributed abundantly in the brain and plays an essential role in potentiating neural network formation in the central nervous system and facilitating neuronal integration in the hippocampus (Van Praag et al., 2005; Erickson et al., 2011; Trinchero et al., 2017). Based on a previous report, BDNF deficiency promoted AD progression, and this important factor is associated with ions dyshomeostasis, neurohormone deficiency, and the appearance of neurotoxins (Zimbone et al., 2018). Extensive molecular pathology studies for AD have reported that aberrant Aβ and phosphorylated Tau trigger neuronal cell death and AD progression. Unfortunately, the etiology of AD is not fully understood, and current symptomatic medications do not completely prevent the progression of AD. Thus, the current study aims to investigate the GAP-43 interaction with BDNF in the hippocampal neuron to develop a therapeutic target for AD. In the current study, we provide molecular evidence demonstrating that the interaction between GAP-43 and BDNF in neurons is associated with the inhibition of aggregated Aβ pathology. Moreover, the 7.8-dihydroxyflavone (DHF) small molecule, which imitates the biological functions of BDNF, triggers GAP-43 protein expression in primary hippocampal neurons and reduces amyloidogenesis.

## Materials and methods

### Primary cultured rat or mouse hippocampal neurons and HT-22 cells

Animal care and handling were performed according to Hallym University Animal care guidelines. Primary rat hippocampus neurons were cultured as previously described (Chen et al., 2021). All rats were purchased from the DLB company. The protocol was reviewed and approved by the Hallym University Institutional Animal Care and Use Committee. HT-22 cells were cultured in DMEM/F12 and 10% FBS and penicillin (100 units/ml)-streptomycin (100 μg/ml) were added (all from Hyclone). Cells were incubated at 37°C in a humidified 5% CO_2_ atmosphere.

### Animals

All animals were housed in filter-topped cages under a 12 h light/dark cycle at an ambient temperature of 22°C. Females and males were kept separate. Tap water and rodent chow were available *ad libitum*. Investigators were blinded to the group allocation during the animal experiments.

### Cell toxicity test

Cell viability was measured colorimetrically using the Cell-TiterBlue (CTB, Promega, Madison, WI, USA) fluorescence-based assay. Cells were plated at a density of 1000 cells/well in 96-well plates (BD Biosciences, San Diego, CA, USA). si GAP-43, si BDNF, and 7.8-DHF were introduced with lipofectamine 3000 to each well after 75% HT-22 cell confluences and then incubated for an additional 2 days. After incubation, 30 μL CTB reagent was added to each well and incubated at 37°C and 5% CO_2_ for 2.5–5 h. The fluorescence of the resorufin product was measured on a FluoDiaT70 fluorescence plate reader (Photon Technology International, Birmingham, NJ, USA). Wells that included vehicle but not protein served as the negative control (0% toxic), and wells containing 10% DMSO served as the positive control (100% toxic). Percent toxicity was calculated using the following equation:


$$\begin{array}{l} \%Toxicity=100-\left[100\left(\left\{S\right\}-\left\{P\right\}/\left\{N\right\}-\left\{P\right\}\right)\right] \end{array}$$

Each independent variable is the average of three plate replicates from the negative control ({N}), positive control ({P}), and samples ({S}). Results presented for viability experiments are an average of three experiments conducted independently on different days. Error bars represent the standard error of the mean (SEM).

### Generation of Aβ 1-42 PFFs

Aβ 1-42 fibrils were prepared in reaction (500 μL per tube) containing 2 mg/mL Aβ 1-42 peptide monomers in PFF reaction buffer (50 mM Tris-HCL, 50 mM NaCl, pH 7.4). The monomer proteins were incubated for several days at 37°C, with orbital shaking at 300 rpm until samples appeared cloudy. Usually, the reactions were subjected to 4–6 days of shaking for PFF generation. PFFs were validated by Th-T *in vitro* assay.

### Th-T assay

Thioflavin T stock solution was prepared by dissolving 16 mg Th-T in 50 mL PBS. The solution was filtered through a 0.2 μm syringe filter (Sigma-Aldrich, cat# T3516). The stock solution was diluted into the PBS to inject the working concentration (2.5 μL of 1 mM Th-T stock solution per each well of the 96-well plate). The monomers and PFFs were added according to the working concentration (10 μM). The total volume per each well was fixed at 100 μL. The rest volume except for Th-T solutions and proteins was made up using phosphate buffer. The reaction plate was excited at 450 nm and emitted at 490 nm to measure the relative fluorescence units on the plate reader (Molecular Devices, SpectraMax iD3, California, USA). Measurements were conducted every 15 min.

### Immunofluorescence

Mice were anesthetized and then transcardially perfused with cold PBS and 4% paraformaldehyde (PFA). The brains were stored for 24 h in 4% PFA at 4°C and then embedded in paraffin. Serial 5-μm-thick sections from all animal groups were processed in parallel for immunohistochemistry and immunofluorescence. Free-floating slices were rinsed in PBS then permeabilized and blocked with PBS-BT [50 mM Tris-HCL, 150 mM NaCl, 3% bovine serum albumin (BSA), 0.1% Triton-X100, pH 7.4] blocking solution for 1 h. Afterward, the sections were incubated with primary antibodies (see Supplementary Table 1) in a 2% normal donkey serum (NDS) and 0.3% Triton X-100 PBS solution on a shaker overnight at 4°C. The next day, sections were rinsed and incubated with corresponding secondary antibodies directly conjugated with fluorophores (1:3000, Cy3, Cy5, and Alexa Fluor 488 conjugate from Jackson ImmunoResearch) for 2 h at room temperature. Finally, slices were rinsed in PBS and mounted (Sigma Aldrich, F4680).

### Immunoblotting

Cells and brain samples were lysed and, if necessary, homogenized in lysis buffer (50 mM Tris, pH 7.4, 40 mM NaCl, 1 mM EDTA, 0.5% Triton X-100, 1.5 mM Na3VO4, 50 mM NaF, 10 mM sodium pyrophosphate, and 10 mM sodium β-glycerophosphate, supplemented with a cocktail of protease inhibitors). Lysates were then centrifuged for 20 min at 4°C 15000 rpm and the protein concentration of the supernatant was measured with the Pierce BCA Protein Assay kit (Part No. 23225). The supernatant was denatured at 95°C in Laemmli buffer. After loading and running proteins in an SDS–PAGE gel, the samples were transferred to a nitrocellulose membrane. Membrane blocking and antibody staining were performed according to the primary antibody manufacturer's instructions. Information on the primary antibodies used to analyze specific molecules can be found in Supplementary Table 2.

### GAP-43 gene-expression profiling dataset

GAP-43 and BDNF gene expression profiling in the hippocampus of patients with AD was conducted using a gene dataset available on the Allen Brain Atlas from the Adult Changes in Thought (ACT) study. Gene expression profiling was done using Affymetrix Human Genome U133 Plus 2.0 GeneChip arrays. We analyzed probe set data for GAP-43 and BDNF in the hippocampus CA3 region and selected the GAP-43 probe set on the criterion of “present” (detectable) call.

### Protein-protein interaction prediction analysis

Protein-protein interaction was analyzed and visualized using ClusPro 2.0 (https://cluspro.bu.edu/) and PyMOL 2.5 (https://pymol.org/2/) software. Protein data were collected from Protein Data Bank (PDB, USA), and PDB files for GAP-43 and BDNF were entered into receptor protein and ligand protein. Binding residues are presented as the technical preset mode and modified with extensive binding atomic groups per residue. Polar contacts, inter-chain contacts, and pi interactions were investigated at the target amino acid regions (Reyes et al., 2013).

### Quantification and statistical analysis

All data were expressed as mean ± S.E.M. from three or more independent experiments. Representative morphological images obtained from at least three experiments with similar results are provided. Image J 1.47 software was used to analyze IF experiments, and Image Lab^TM^ software was used for western blots analysis. The statistical analysis of results was performed using GraphPad5 (Prism) software. All data were tested for normal distribution in order to analyze results accordingly using parametric or non-parametric tests. To compare results between two groups, Student's unpaired *t*-test was used. When more than two groups were compared, one-way ANOVA followed by the Tukey *post hoc* test was applied. For repeated measures, a Repeated-Measures (RM) ANOVA or 2-way ANOVA test was performed followed by Tukey multiple comparisons *post hoc* test. Assessments with a *p*-value of < 0.05 were considered significant.

### Stereotaxic injection and treatments

Stereotaxic coordinates were determined according to the rat brain atlas of Paxinos and Watson (1997) and the mouse brain atlas of Paxinos and Franklin (2001). A total of 2 μg of Aβ PFFs was injected in the hippocampus brain region of *C57BL/6J* mice to generate the Aβ AD patho-molecule dependent AD mouse model. Aβ 1-42 peptide was purchased from Sigma-Aldrich (Cat.AG968 respectively). Three-month-old *C57BL/6J* mice were anesthetized with isoflurane. Meloxicam (5 mg/kg) was injected subcutaneously for analgesia (Loxicom, Norbrook, USA). Unilateral stereotaxic injection of Aβ PFFs or control Aβ monomer was performed at coordinates corresponding to the hippocampus: anteroposterior (A/P): −2.5 mm, mediolateral (M/L): +/−2.0 mm from Bregma, and dorsoventral (D/V): 1.8 mm from dura surface. A total of 2μl PFFs (1μg/μl) was injected at a rate of 0.25 μl/min using a 10 μl Hamilton syringe. The needle was kept in place for 5 min after the injection was completed and then gently removed. Mice were placed on a heating pad until they recovered from the anesthesia.

### Real-time RT-PCR

Total mRNA was isolated from si GAP-43 or si BDNF or both GAP-43 and BDNF knock-down with 7.8-DHF compounds treated cell lysates using TRIzol reagent (Invitrogen). The complementary DNA was synthesized using the iScript cDNA synthesis kit (Bio-Rad). Quantitative PCR was performed on a LightCycler 480 real-time PCR system using LightCycler 480 SYBR Green a Master Mix (Roche). The following target-specific primer was used: GAP-43: (F): 5'- AGGAAAGGAGAGAAGGCAGG-3', (R) 5'-GCAGGAGAGACAGGGTTCAG-3'. GAPDH: (F): 5'-TGCTTCACCACCTTCTTGA-3', (R): 5'-TCACCATCTTCCAGGAGC-3'; GAPDH : (F): 5'- GTCTCCTCTGACTTCAACAGCG3', (R) 5'-ACCACCCTGTTGCTGTAGCCAA-3'. The relative gene expression was normalized to GAPDH expression and assessed using the 2-ΔΔCt method.

### Immunoprecipitation

Aβ PFFs- or monomer-injected mouse hippocampus brain tissues were lysed in lysis buffer (50 mM Tris-HCl (pH 7.4), 150 mM NaCl, 1 mM EDTA, 1% Triton x-100, 0.1% SDS, 0.5% Sodium deoxycholate) with phosphatase inhibitors (Roche PhosSTOP) and complete protease inhibitor mixture cocktail (Sigma-Aldrich). The lysates were incubated with the BDNF antibody (Rabbit monoclonal, Cat: ab108319, 500:1) for 1 h at 4°C. Next, 20 μl Protein A/G Plus-Agarose (Santa Cruz Biotechnology) was added and incubated overnight at 4°C on a rocker platform. The immunoprecipitates were collected by centrifugation at 800 rpm for 5 min at 4°C and washed five times with lysis buffer. The samples were boiled in an SDS loading buffer for 3 min and subjected to immunoblotting assay using an antibody against GAP-43.

## Human brain tissue samples of a patient with AD

All brain tissues were obtained from the Emory Alzheimer's Disease Research Center (ADRC) Brain Bank. Human post-mortem tissues were acquired under proper Institutional Review Board (IRB) protocols. AD was diagnosed according to the criteria of the Consortium to Establish a Registry for AD and the National Institute on Aging. Diagnoses were further confirmed by the presence of amyloid plaques and neurofibrillary tangles in formalin-fixed tissue. Ages and post-mortem times were similar between patients with AD and controls. Informed consent was obtained from the subjects. The study was approved by the Biospecimen Committee.

### Morris water maze test

The Morris water maze test was used to detect the spatial learning and memory of the mice with AD, as described previously (Zhang et al., 2014). Mice were trained for four trials per day for 7 consecutive days. The mice were allowed to search for the platform for 60 s. Otherwise, they would be guided to the platform manually and were allowed to stay on it for 15 s before the next trial. After the trials, the mice were dried and put back in the cage. After the last training day (day 7), a spatial probe trial was performed. The platform was removed and the mice were allowed to swim for 60 s. The percentage of time spent in the platform quadrant was measured. All trials were recorded by a computerized tracking system that analyzed the distance the mice moved, the latency required to reach the platform, and the swim speed using ANY-Maze software (San Diego Instruments).

### Novel objection recognition test

For habituation, mice were placed in the open field and each mouse was allowed to freely explore for 5 min. The next day, two different objects were put in opposite corners of the open field, and mice were placed in the middle of the open field and allowed to explore for 10 min. For the analysis, one of the objects used during training was replaced with a novel object in the opposite corner. Mice were placed in the middle of the field and allowed to explore for 10 min. Time and touch numbers in exploring the novel object were analyzed and the discrimination index was calculated.

## Results

### GAP-43 and BDNF are significantly reduced in the hippocampus brain region of patients with AD

**Figure 1:**
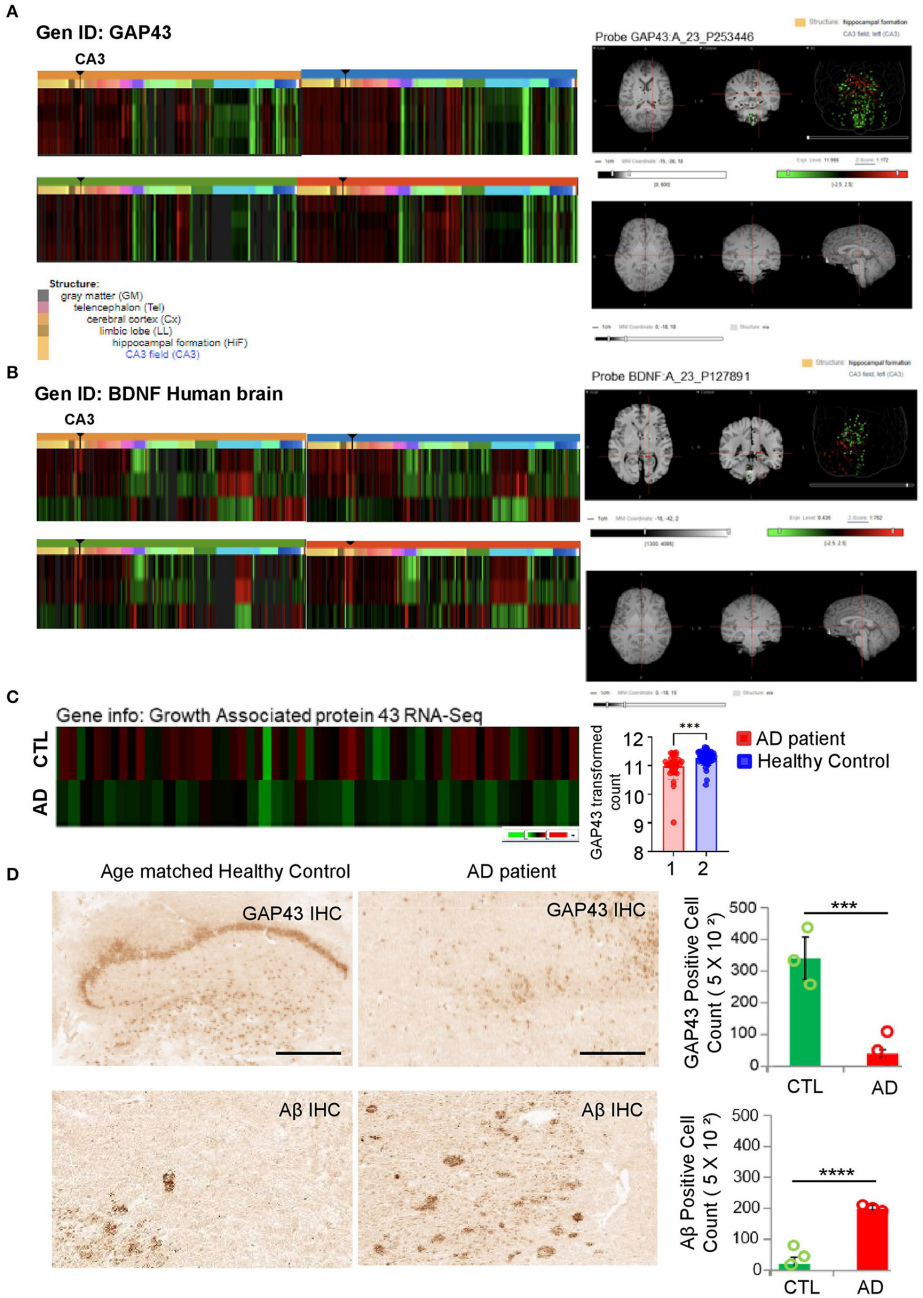
Investigation of gene level among GAP-43 and BDNF from patients with AD and age-matched healthy controls. **(A, B)** GAP-43 or BDNF mRNA expression microarray data from four healthy adults' hippocampus brain regions. Microarray data were obtained from the Allen Human Brain Atlas. Gene codes at the upper left side of the heatmap are probes used in the analysis. Right-hand side panels show representative MRI images of the GAP-43 or BDNF expression level in the hippocampus CA3 region, conducted with the matched probe. **(C)** CTL vs. GAP-43 mRNA expression level of patients with AD from the Adult Changes in Thought (ACT) study (left panel). GEO database set ID: GDS4758/8081810, post-mortem Alzheimer's disease brains: Hisayama study (right panel). **(D)** Immunohistochemistry analysis of amyloid patho-molecule protein and GAP-43 expression levels conducted using GAP-43 and Aβ antibodies (scale bar, 100 μm). Specific antibody positive cell quantification bar graphs of GAP-43 and Aβ are shown in the right-hand side panels. Data are shown as mean + SEM. Statistical significance was determined by an unpaired *t*-test. *N* = 3 for each group. ^***^P < 0.00005; ^****^P < 0.00001.

Previous studies have indicated an association between amyloidogenesis and GAP-43 or BDNF in CSF samples of patients with AD (Sandelius et al., 2019; Mahaman et al., 2021; Simrén et al., 2023). A recent study reported that GAP-43 and BDNF were reduced in 5XFAD AD mice expressing five AD-linked mutations (human APP of Swedish, Florida, and London; PSEN1 of M146L, and L286V) with every-other-day feeding (EOD) (Lazic et al., 2020). To address the importance of GAP-43 and BDNF in the hippocampus brain region, we confirmed the expressions of GAP-43 and BDNF in the human hippocampal formation, especially in CA3, which is associated with AD progression (Figures 1A, B). We also applied the GAP-43 gene ID to the Allen Brain Atlas aging and dementia data portal to analyze the GAP-43 mRNA with AD progression. Consistently, we identified GAP-43 mRNA reduction in the hippocampus region of patients with AD (Figure 1C and Supplementary Figure 1). Moreover, we performed GAP-43 immunohistochemistry using the age-matched healthy control or the brain sections of a patient with AD and observed a reduction of GAP-43, which is consistent with the Allen Brain Atlas database (Figure 1D). Also, GAP-43 is widely expressed with BDNF in the whole brain regions in mice including the parts in charge of memorial function, which correlates with the human database (Supplementary Figure 2).

### Aβ PFFs induced hippocampal neuronal cell toxicity *via* GAP-43 and BDNF deprivation in primary hippocampus neurons

**Figure 2:**
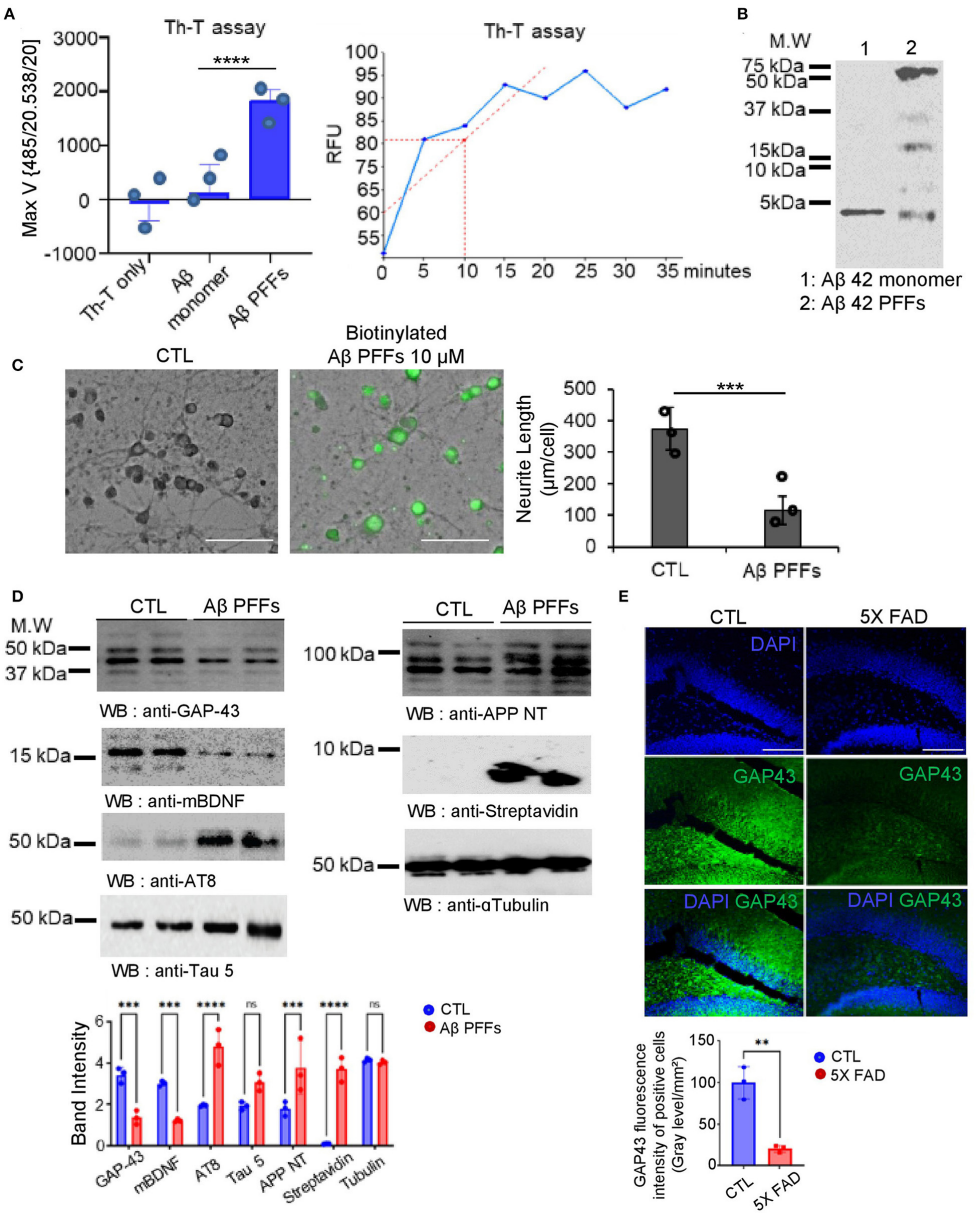
Aβ 1-42 pre-formed fibrils (PFFs) structural analysis with neurotoxicity effects. **(A)** Thioflavin T compound assay showed different aggregation RFU rates of Aβ 1-42 peptide. **(B)** SDS-PAGE coomassie blue gel analysis for Aβ 1-42 PFFs on the final reaction day. **(C)** Representative images of mouse hippocampal neuron cells following 24 h incubation with PBS (CTL) or biotinylated Aβ 1-42 PFFs (10 μM) (scale bar, 40 μm). Right side, a quantification bar graph of neurite length (μm/cell). Data are shown as mean + SEM. Statistical significance was determined by an unpaired *t*-test. *N* = 3 for each group. ^***^P < 0.00005. **(D)** Immunoblotting assay of GAP-43, mature BDNF, AT8, Tau 5, APP NT, streptavidin, and alpha-tubulin levels in control vs. Aβ 1-42 PFFs (10 μM)-treated primary neuron cell lysates (*N* = 3). Band intensity quantification bar graph (bottom). ^***^P < 0.001, ^****^P < 0.0001, ns; not significant. **(E)** Immunofluorescence staining analysis under amyloid genetic effects in 5X FAD (7 month old) mouse brain hippocampus to observe the GAP-43 reduction (scale bar, 20 μm). Error bars represent the mean ± SEM. Statistical significance was determined using a one-way ANOVA followed by a *post hoc* Tukey test for multiple-group comparison. ^**^P < 0.01, ^***^P < 0.001, ^****^P < 0.0001, ns, not significant.

We verified that GAP-43 is well expressed in mature hippocampal neurons but inversely expressed with AD pathogenesis. Presumably, the molecular mechanism triggering Aβ aggregation or misfolding might involve the reduction of GAP-43 and BDNF in the primary hippocampus neurons to induce AD pathologies. To demonstrate this hypothesis, we generated the Aβ 1-42 preformed fibrils (PFFs), which are used as the pathological molecular model for aberrant fibrils of AD. To confirm the Aβ PFFs structure, we performed the thioflavin T (Th-T) Aβ fibrillization kinetic assay comparing monomer Aβ 1-42 peptide and showed that Aβ 1-42 PFFs formed the aggregated fibers in a reaction time-dependent manner (Figure 2A) based on SDS-PAGE analysis (Figure 2B). After that, we performed Aβ PFFs biotinylation to determine Aβ PFFs neuronal toxicity using a live cellular imaging technique. We observed primary hippocampal neuronal cell death and neurite length change in the Aβ PFFs-treated group, with biotin fluorescence (Figure 2C). In addition, we made primary hippocampal neuronal cell lysates to investigate the molecular signal transduction related to AD pathologies. Consistently, we observed BDNF and GAP-43 reduction in Aβ PFFs-treated group, and inversely, the Tau and APP protein expressions increased (Figure 2D). Interestingly, we identified a decrease of GAP-43 in the hippocampus brain region of a 6-month-old 5X FAD Tg mouse but not in the control mouse (Figure 2E).

### GAP-43 stimulation through 7.8-DHF and the interaction with BDNF under Aβ PFFs stress

**Figure 3:**
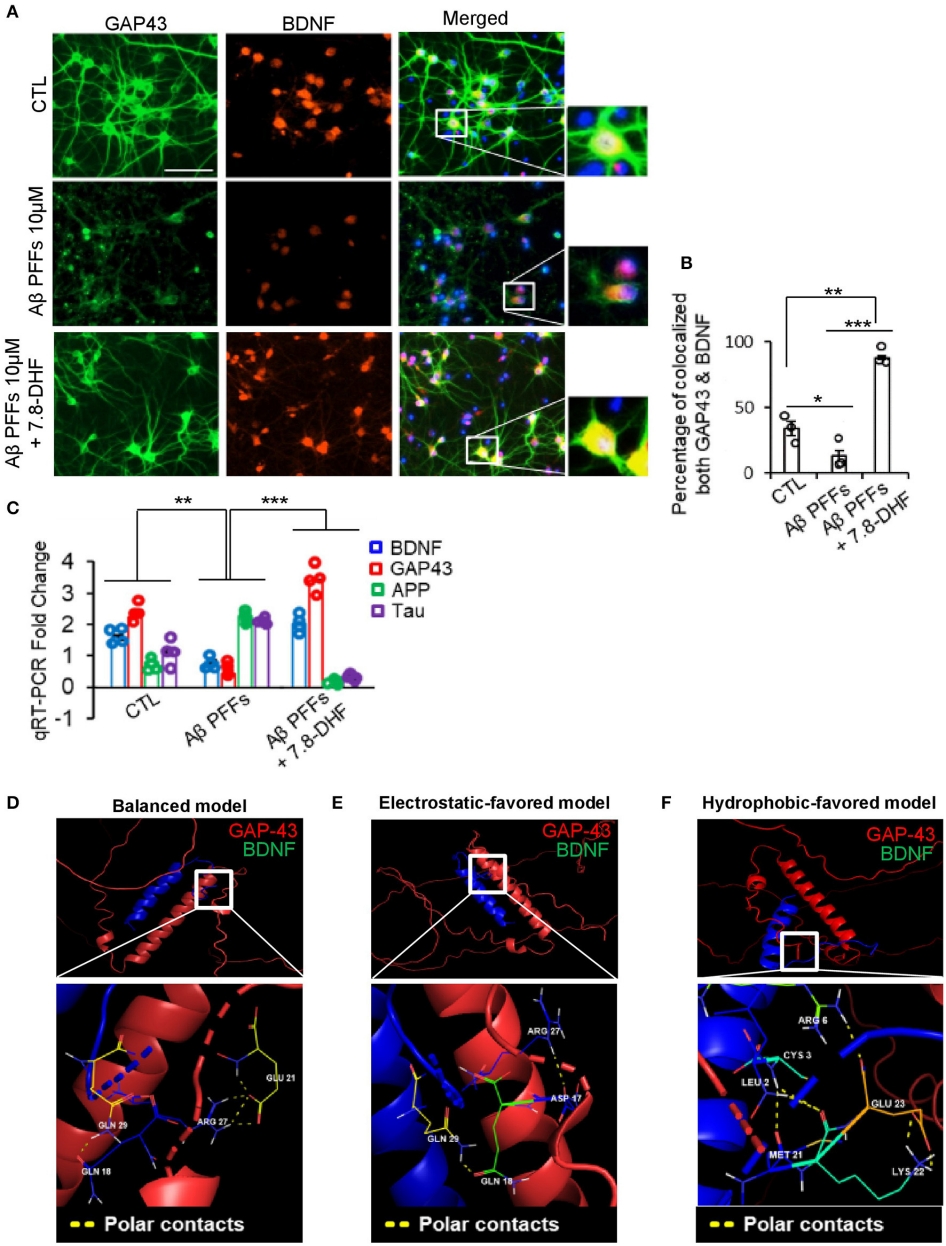
GAP-43 and BDNF co-localized in primary hippocampal neurons *via* direct interaction. **(A)** Co-immunofluorescence staining analysis of Aβ monomer or Aβ 1-42 PFFs or Aβ 1-42 PFFs + 7.8-DHF treated primary hippocampus neurons conducted using BDNF/GAP-43 antibodies (scale bar, 50 μm). **(B)** Percentage of co-localized BDNF/GAP-43 quantification bar graph. Error bars represent the mean ± SEM. Statistical significance was determined using a one-way ANOVA followed by a *post hoc* Tukey test for multiple-group comparison. ^*^P < 0.05; ^**^P < 0.01; ^***^P < 0.001. **(C)** qRT-PCE analysis bar graph (blue: BDNF, red: GAP-43, green: APP, and purple: Tau) of Aβ monomer or Aβ 1-42 PFFs or Aβ 1-42 PFFs + 7.8-DHF treated primary hippocampal neurons. All real-time PCR experiments were repeated at least four times. Error bars represent the mean ± SEM. Statistical significance was determined using two-way ANOVA followed by a *post hoc* Bonferroni test for multiple-group comparison. ^**^P < 0.01; ^***^P < 0.001. **(D–F)** Predictive three-dimensional model of direct interaction structures between GAP-43 (PDB ID: AF-O95631-F1) and BDNF (PDB ID: AF-P37840-F1) obtained by using ClusPro 2.0 and PyMOL 2.5 software analysis. The balanced model, electrostatic-favored model, and van der Waals force-favored model showed the polar contacts between GAP-43 and BDNF.

We examined the co-localization of GAP-43 and BDNF in the presence of Aβ PFFs *via* co-immunofluorescence staining. Strikingly, GAP-43 and BDNF co-localized in the hippocampus neurons and the small compound 7.8-DHF mimicked BDNF function (Figures 3A, B). Quantitative RT-PCR (qRT-PCR) demonstrated that the mRNA augmentation of APP and Tau in the hippocampal neuronal cell (HT-22 cell) by Aβ PFFs was inversely coupled with GAP-43 and BDNF mRNA but this was not the case in the 7.8-DHF-administered group (Figure 3C). Furthermore, we investigated the predictive interacting sites between GAP-43 and BDNF proteins. We obtained a three-dimensional structure model of both proteins, including the predictive interaction sites, using ClusPro 2.0. As a result, three types of structures (balanced, electrostatic-favored, and van der Waals force-favored) showed the polar contacts between netrin-1 and the 124th−135th amino acids in the C-terminus of BDNF (Figures 3D–F).

### Deletion of GAP-43 or BDNF in the HT-22 cells induces ROS and caspase 3 activity

**Figure 4:**
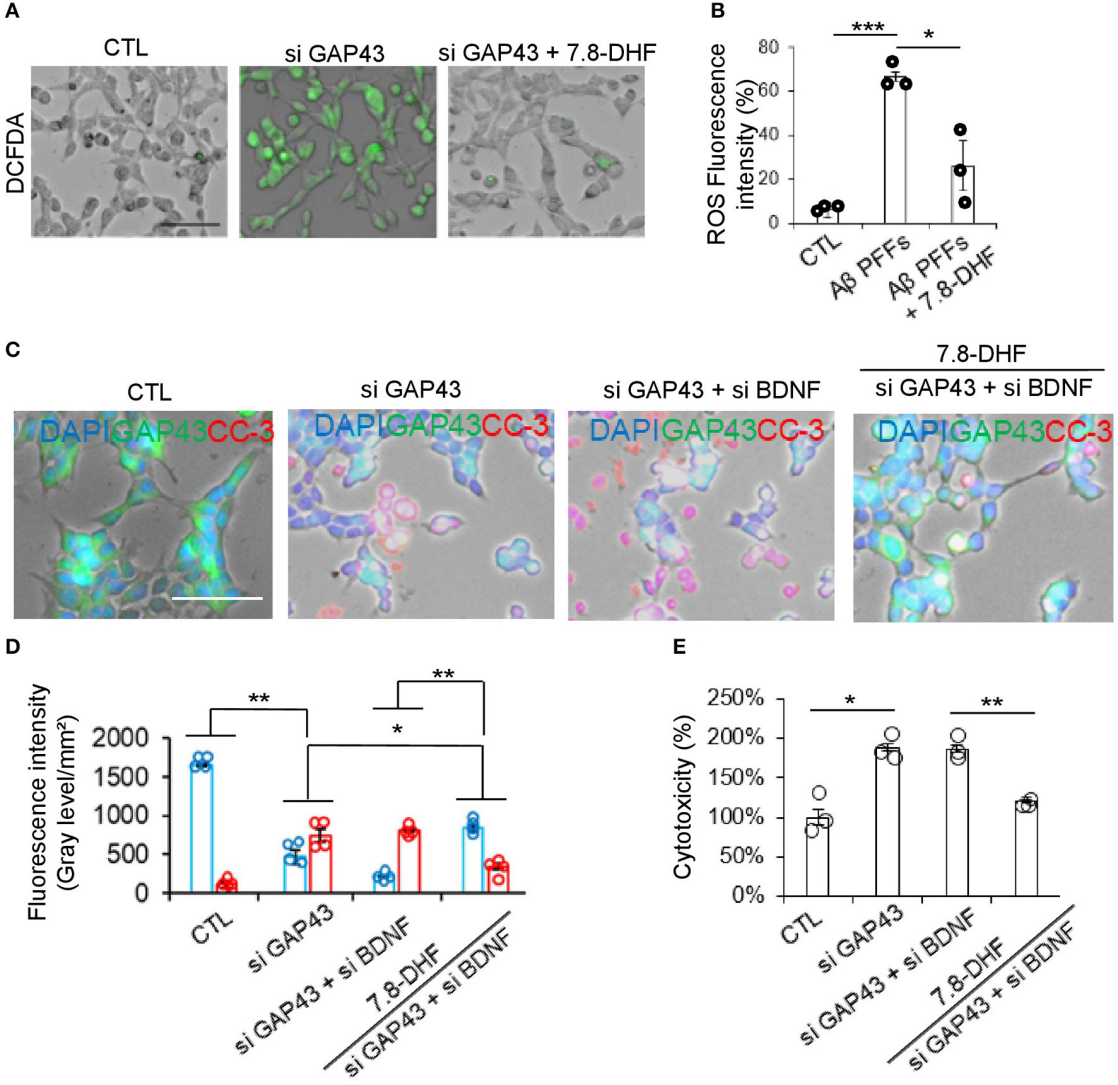
Deletion of GAP-43 or BDNF with or without 7.8-DHF in the HT-22 cell induced ROS and caspase-3 activity. **(A, B)** ROS levels in HT-22 cells after si GAP-43 or si BDNF treatment with or without 7.8-DHF. Scale bar: 60 μm. ROS fluorescence intensity bar graph data are mean ± SEM (^*^P < 0.05;^***^P < 0.001). **(C)** Immunofluorescence co-staining of GAP-43 (green) and cleaved caspase-3 (red) in the HT-22 cells with si CTL or si GAP43 or si BDNF or si GAP43 + si BDNF or si GAP43 + si BDNF + 7.8-DHF treated groups. **(D)** Quantification bar graph (blue: GAP-43, red: cleaved caspase-3). *N* = 4. **(E)** Cytotoxicity analysis bar graph. *N* = 3, error bars represent the mean ± SEM. Statistical significance was determined using a one-way ANOVA followed by a *post hoc* Tukey test for multiple-group comparison. ^*^P < 0.05; ^**^P < 0.01.

We investigated whether the loss of function of GAP-43 or BDNF induces hippocampal cell death through ROS and caspase-3 activation. We determined intercellular ROS levels with DCFDA fluorescent dye. Quantitative analysis revealed that the knock-down of GAP-43 significantly increased ROS but not in the si GAP-43 + 7.8-DHF group (Figures 4A, B). Subsequently, we conducted caspase-3 activity in CTL, si GAP-43, si GAP-43 + si BDNF, and si GAP-43 + si BDNF+ 7.8-DHF groups. The highest caspase-3 activity was observed in the si GAP-43 + si BDNF group. However, 7.8-DHF re-stimulated the GAP-43 and BANF protein expression (Figures 4C, D). Then, we performed the LDH assay for cellular toxicity analysis. Remarkably, cytotoxicity levels were substantially reduced in the si GAP-43 + si BDNF 7.8-DHF group; however, the levels inversely increased in knock-down GAP-43 or BDNF groups (Figure 4E).

### GAP-43 and BDNF are reduced in AD mouse model with memory defects

**Figure 5:**
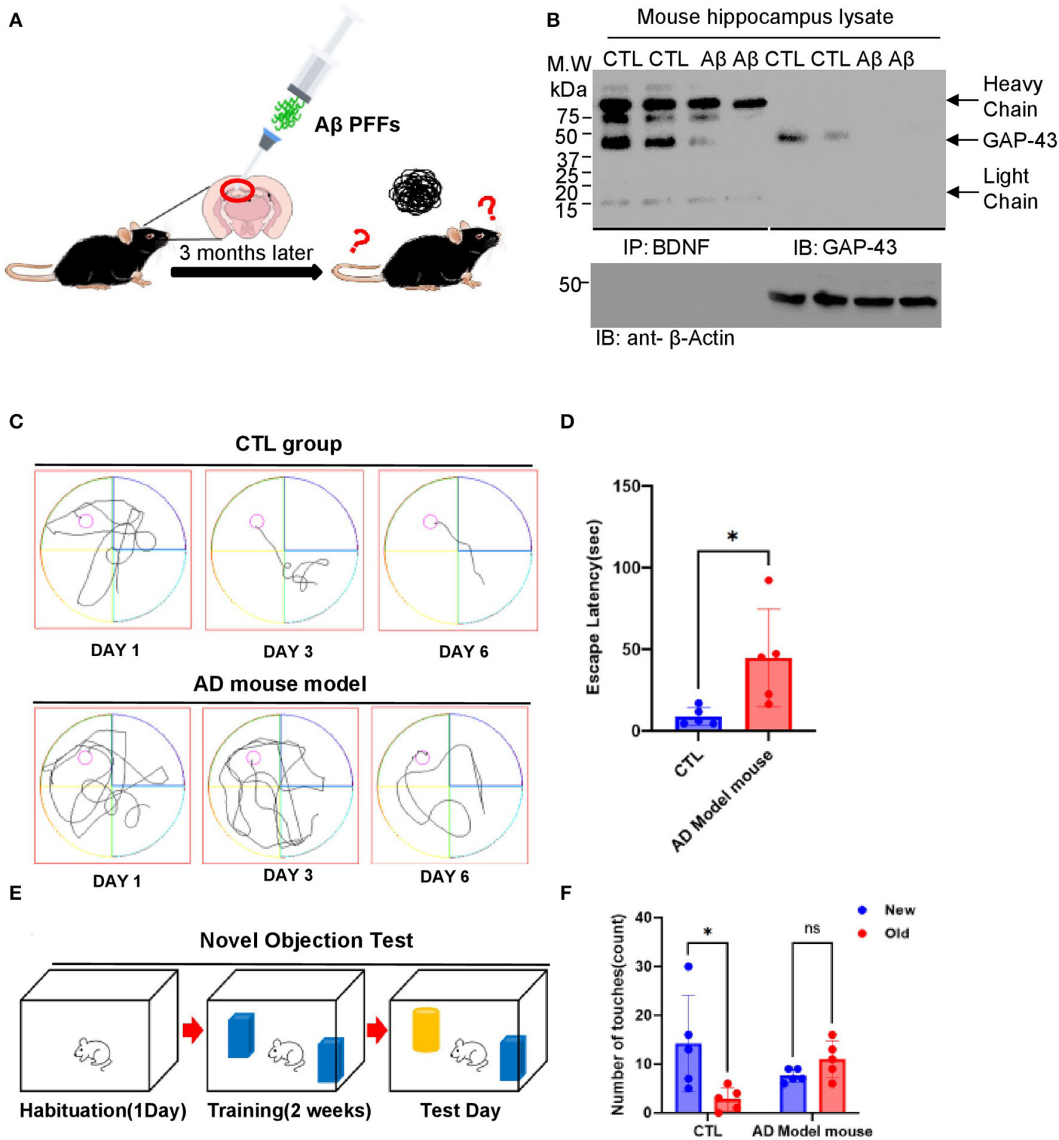
GAP-43/BDNF tightly interacts in the mouse Aβ 1-42 PFFs preclinical model for AD. **(A)** Diagram of AD mouse model generation. **(B)** GAP-43 strongly interacts with BDNF. IP assay performed using Aβ PFFs-injected mouse's hippocampus brain lysates. **(C, D)** Spatial memory assessed by the Morris water maze test. The distance traveled to the platform by mice injected with PBS or Aβ PFFs is shown. Integrated time traveled in the water maze test. **(E, F)** Schematic of the novel object recognition test protocol **(E)** and the time spent exploring the familiar and novel objects **(F)**. *N* = 5 mice in the CTL group and Aβ PFFs-injected group. Individual values and mean ± SEM are shown, N>5 animals in each group. Tukey *post hoc* analysis after one-way ANOVA, ^*^P < 0.05 compared to the CTL group.

GAP-43 is greatly downregulated in human and AD mouse models (Greco et al., 1992; Cheetham et al., 1996; Nishal et al., 2023). GAP-43 and BDNF reduction in aged 5XFAD mice accelerates AD pathologies and triggers cognitive deficits. To investigate the roles of GAP-43 and BDNF and their involvement with memory dysfunction, we conducted immunoprecipitation analysis using AD mouse brain lysates. The diagram in Figure 5A presents the generation step of the Aβ PFFs-injected AD mouse model. Consistently, immobilized BDNF strongly interacted with GAP-43 in AD mouse hippocampus tissue lysates (Figure 5B). Next, we examined the effect of cognitive deficits *via* water maze and novel object testing in the Aβ PFFs-injected mouse model. The Aβ PFFs-injected group showed memory dysfunction associated with the expression levels of GAP-43 and BDNF (Figures 5C–F).

## Discussion

**Figure 6:**
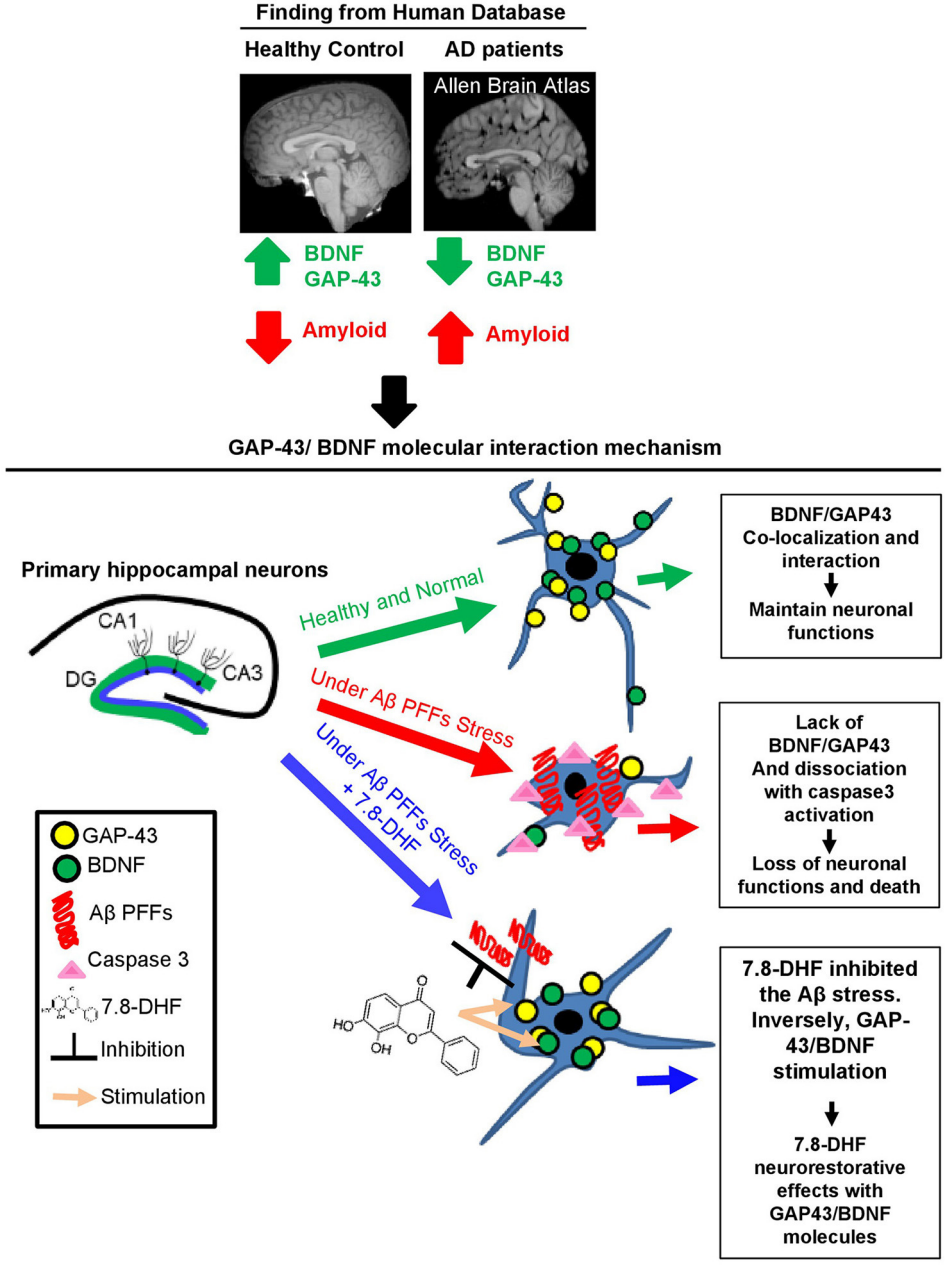
Schematic model of GAP-43/BDNF and Aβ in Alzheimer's disease pathogenesis molecular mechanism. Diagram of GAP-43/BDNF and 7.8-DHF synergic effect and the molecular mechanism in hippocampal neurons.

GAP-43 is related to presynaptic neuronal outgrowth and neuronal plasticity in general. This molecule's first exon encodes the membrane targeting domain and its second exon encodes a calmodulin-binding domain and a protein kinase C (PKC) phosphorylation site, while the 5'- flanking sequence detects initiation of RNA transcription (Holahan, 2015). Brain-derived neurotrophic factor (BDNF)/TrkB triggers downstream of synaptic pathways, including PKC, essential for synaptic functions (Parveen et al., 2019). Therefore, we demonstrated BDNF/GAP-43 direct interaction in hippocampal neurons and AD mice. In the current study, we have identified that GAP-43 interacts with BDNF, which enhances Tau and Aβ molecular pathology in Aβ PFFs-administered AD cellular models. GAP-43 (arginine27 and aspartic acid 17) directly binds with the BDNF (glutamine 29 and 18) and triggers amyloid aggregation and Tau hyperphosphorylation. Notably, in the mature human brain, GAP-43 and BDNF are mainly expressed in the hippocampus, which is associated with the development of AD. Targeting AD pathologies underlying the vulnerability of hippocampal neurons by amyloid-specific deprived molecules might be one of the possible therapeutic approaches to delay or halt AD progression. GAP-43 in cerebrospinal fluid (CSF) has previously been shown to be increased in AD and was correlated with the magnitude of Aβ plaques in the hippocampus, amygdala, and cortex, but was not associated with the α-synuclein or TDP-43 neuropathic molecules (Sandelius et al., 2019; Qiang et al., 2022; Zhu et al., 2022), supporting the notion that GAP-43 might be a marker for hippocampal neuronal cell degeneration, which are most vulnerable to AD pathogens. BDNF/TrkB neurotrophic activation is important in synaptic plasticity, neuronal survival, and hippocampal neuronal functions (Minichiello, 2009). Moreover, in a previous report on the 3XTg/BDNF +/– animal model, BDNF was decreased by half, but neurotrophic signalings were relatively intact (Wang et al., 2019). Interestingly, 3XTg/BDNF +/– mice display similar levels of amyloid and Tau pathologies to 3XTg/BDNF +/+ (Castello et al., 2012). Therefore, this result suggests that chronic reduction of BDNF does not aggravate the triggering of amyloid and Tau pathologies and instead supports the decreased BDNF levels that are found in patients with AD as a consequence of these pathologies. In addition, p-TrkB molecular signaling is decreased in 5X FAD/TrkB +/– mice; there was no difference between these mice and the 5XFAD/TrkB +/+ control mice group in terms of Aβ concentration, aggregated form, or β-amyloidogenic processing of amyloid precursor protein (Yamagishi et al., 2015). However, it is unclear whether BDNF/TrkB signaling invites another molecular factor in hippocampal neurons during neuronal cell death with Aβ production. We have analyzed the genetic effects of the knock-down of GAP-43, BDNF, or both GAP-43/BDNF in primary hippocampus neurons and HT-22 cells. The knock-down group of both GAP-43 and BDNF showed the worst hippocampal neuronal cell toxicity, ROS production, and decrease in neurite length. Strikingly, the BDNF mimetic compound-treated group showed much less neuronal cell death and ROS production as a result of the escalation of GAP-43 mRNA and decreased caspase-3 activation in hippocampal neurons. In addition, the GAP-43 protein expression was reduced during the natural aging process in the hippocampus of 5X FAD mice (Elder et al., 2021). Also, using ClusPro 2.0 software, the BDNF/GAP-43 direct binding sites predict the synergy effect of BDNF and GAP-43 in the primary neurons and co-localization in the hippocampus of mice with AD. Currently, our study shows the physical interaction between GAP-43 and BDNF in human and mouse hippocampal neurons, involving Aβ and Tau molecular signaling with TrkB receptor activation *via* 7.8-DHF. Conclusively, our findings provide new insights into the BDNF/GAP-43 molecular pathway and its potential contribution to AD pathologies (Figure 6).

## Data availability statement

The original contributions presented in the study are publicly available. The data used in the current paper can be found in the respective details listed below:

- GEO database: [https://www.ncbi.nlm.nih.gov/geo/query/acc.cgi?acc=GSE36980]
- Protein-protein interaction: [https://cluspro.bu.edu/login.php / GAP-43 (PDB ID: AF-O95631-F1) and BDNF (PDB ID: AF-P37840-F1)]
- Allen Brain Map: [https://portal.brain-map.org/, https://aging.brain-map.org/donors/summary / GAP-43: M25667.1].

## Ethics statement

The animal study was reviewed and approved by the Animal care and handling were performed according Hallym University Animal care guidelines. Animal care and handling was performed according to NIH animal care guidelines and Emory Medical School guidelines. Written informed consent was obtained from the individual(s) for the publication of any potentially identifiable images or data included in this article.

## Author contributions

EA and SS developed the rationale and designed the experiments, analyzed the data, and wrote the manuscript. YL and EA performed most of the experiments and data analysis. EA and SK performed studies on 5XFAD Tg mice experiments. YL, EK, YJ, BK, SL, and YK provided technical assistance. SK assisted with data analysis and interpretation and critically read the manuscript. All authors contributed to the article and approved the submitted version.

## Supplementary material

Supplementary Figure 1 Information of patient with AD. **(A)** Allen Human Brain Atlas AD patient's information table with Braak stage. **(B)** GAP-43 molecular interaction map obtained from the Sino Biological database.

Supplementary Figure 2 GAP-43 and BDNF are well expressed in the hippocampus, showing molecular interactions. **(A)** Mouse brain dataset analysis gene ID and mouse strain information table. **(B)** *In situ* hybridization (ISH) images and visualized BDNF or GAP-43 expressed regions in the midbrain of C57BL/6J. **(C)** Hippocampal formation (HPF), olfactory areas (OLF), and isocortex brain regions ISH representative images conducted by BDNF/GAP-43 (upper), and quantification bar graph (bottom).

Supplementary Table 1 Key resources information.

Supplementary Table 2 Abbreviation information table.
