# Supplementary figures and images for: GAP-43 closely interacts with BDNF in hippocampal neurons and is associated with Alzheimer's disease progression
Source: Front Mol Neurosci. 2023 Apr 18;16:1150399. doi: 10.3389/fnmol.2023.1150399 (PMC10152972; doi:10.3389/fnmol.2023.1150399)

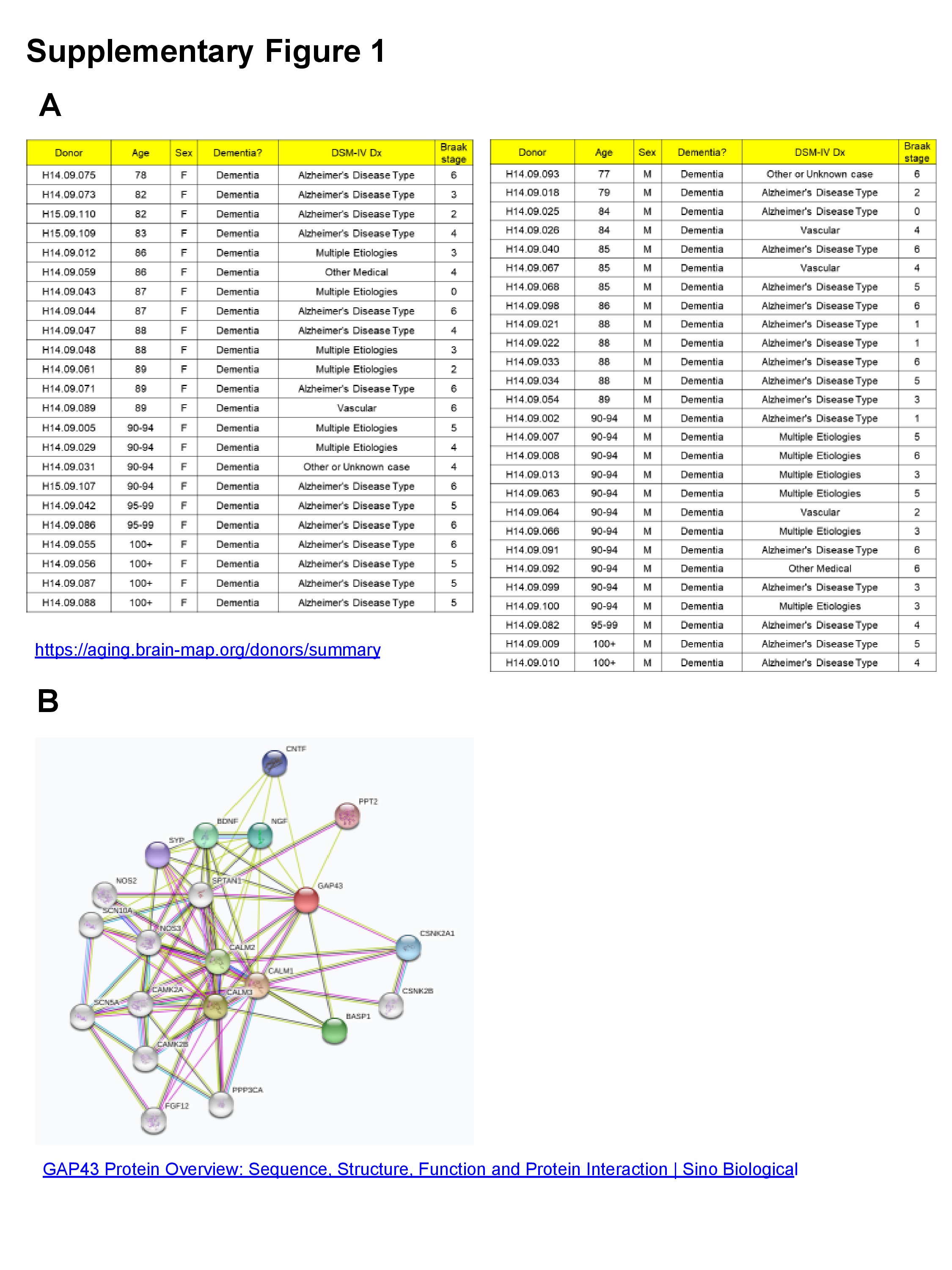

Supplement: Supplementary Figure 1 — Information of patient with AD. (A) Allen Human Brain Atlas AD patient's information table with Braak stage. (B) GAP-43 molecular interaction map obtained from the Sino Biological database. [file Image_1.JPEG]

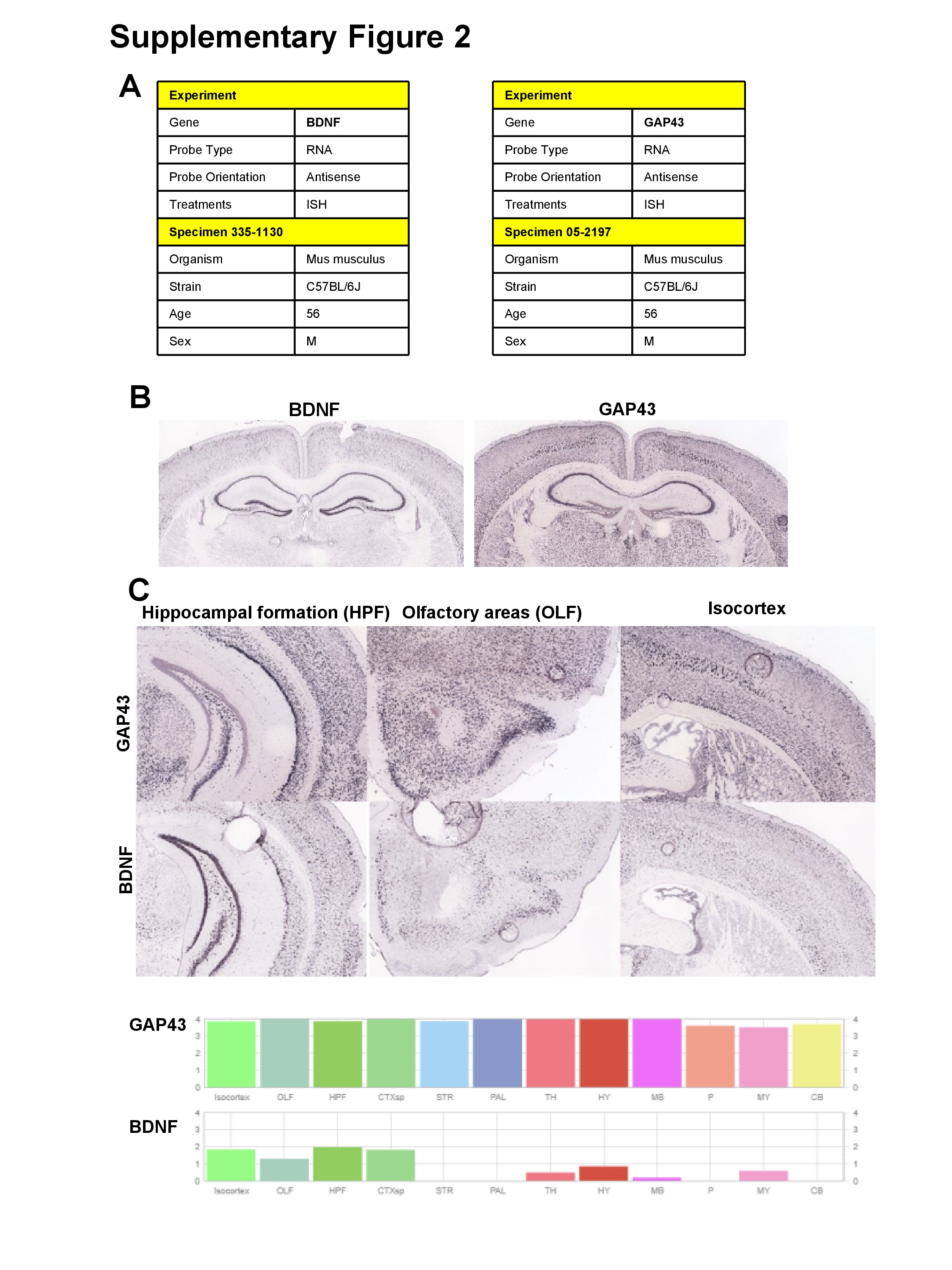

Supplement: Supplementary Figure 2 — GAP-43 and BDNF are well expressed in the hippocampus, showing molecular interactions. (A) Mouse brain dataset analysis gene ID and mouse strain information table. (B) In situ hybridization (ISH) images and visualized BDNF or GAP-43 expressed regions in the midbrain of C57BL/6J. (C) Hippocampal formation (HPF), olfactory areas (OLF), and isocortex brain regions ISH representative images conducted by BDNF/GAP-43 (upper), and quantification bar graph (bottom). [file Image_2.JPEG]

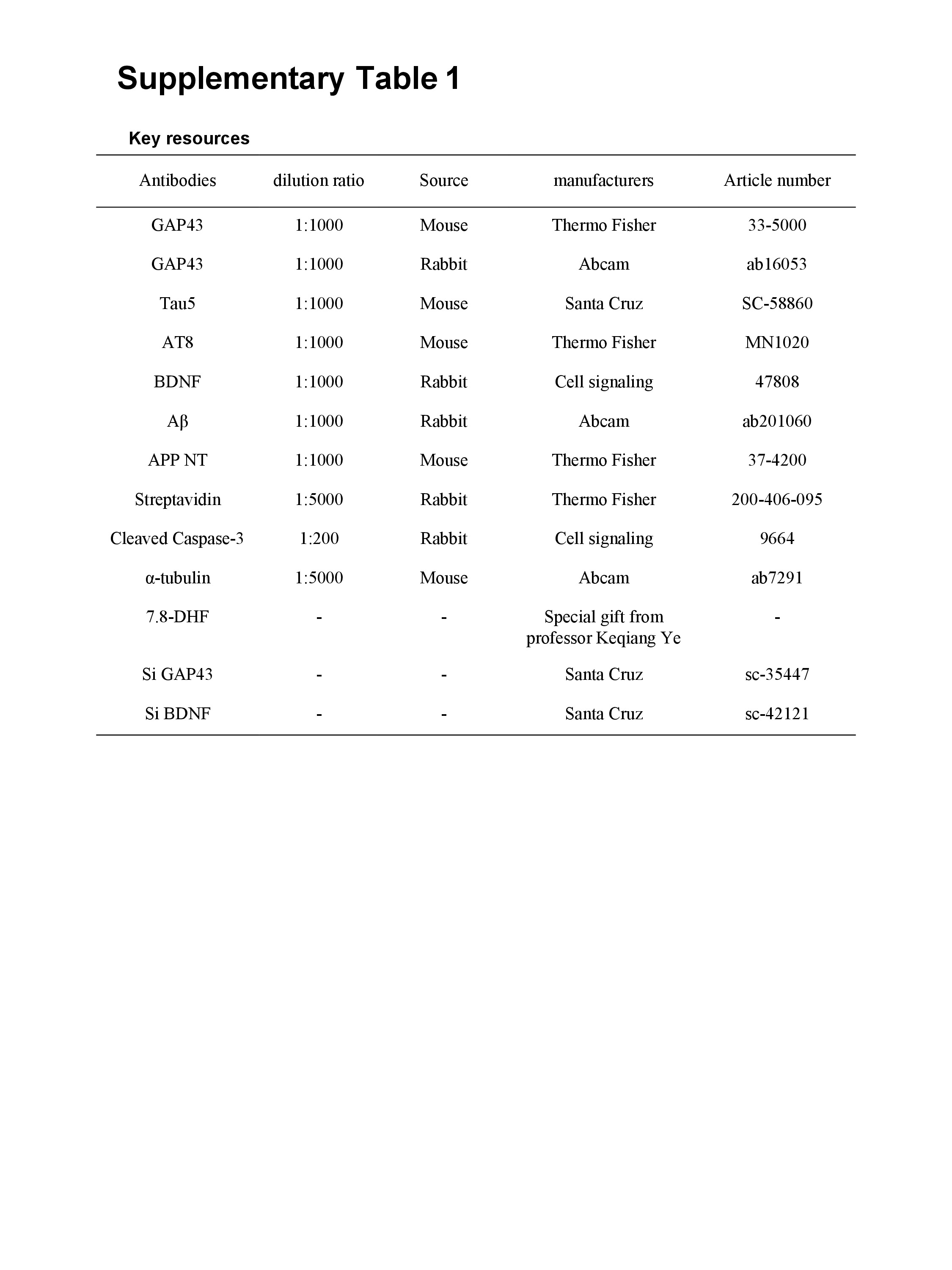

Supplement: Supplementary Table 1 — Key resources information. [file Image_3.JPEG]

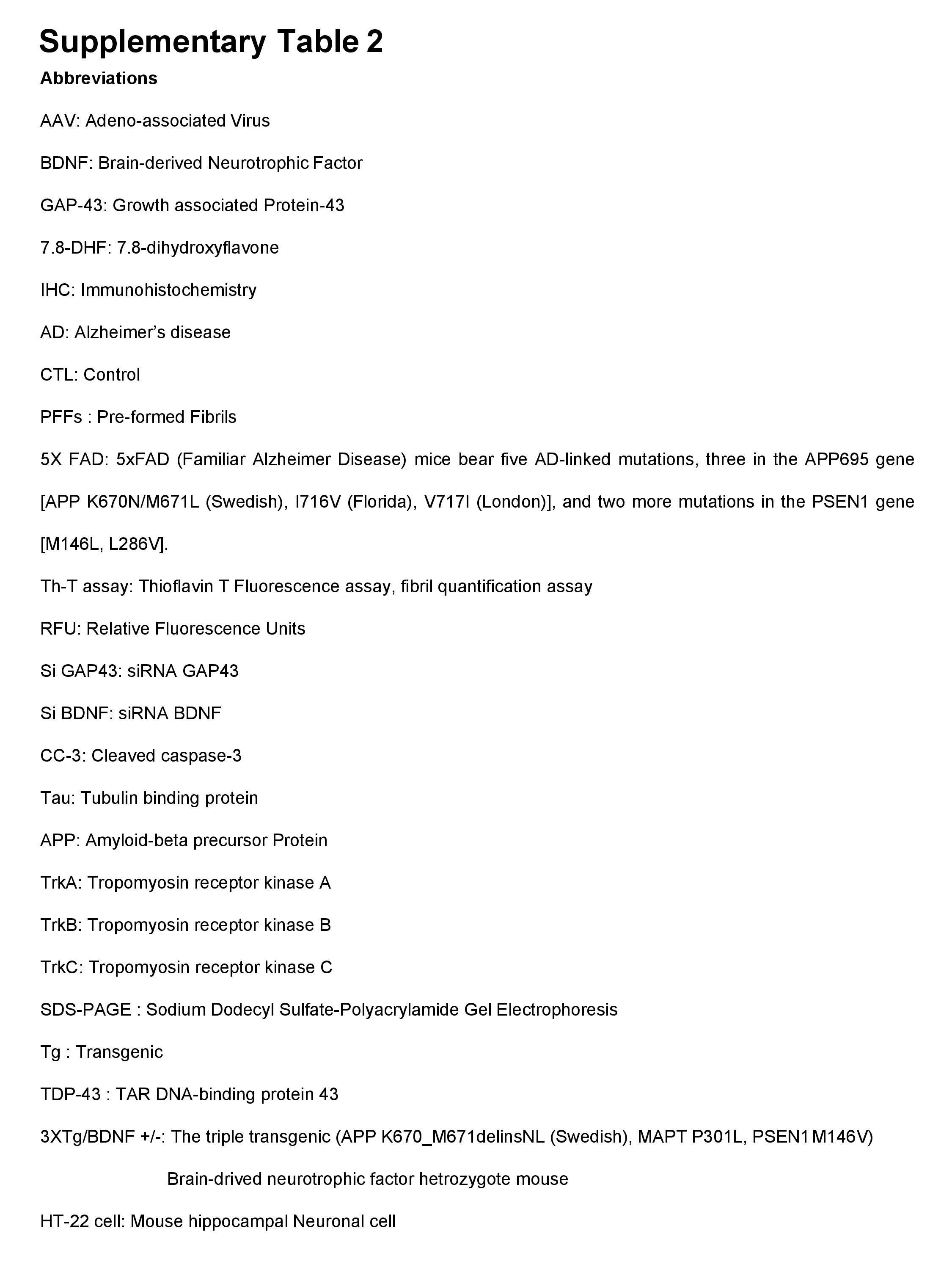

Supplement: Supplementary Table 2 — Abbreviation information table. [file Image_4.JPEG]
